# Screening of an FDA-Approved Drug Library with a Two-Tier System Identifies an Entry Inhibitor of Severe Fever with Thrombocytopenia Syndrome Virus

**DOI:** 10.3390/v11040385

**Published:** 2019-04-25

**Authors:** Shuofeng Yuan, Jasper Fuk-Woo Chan, Zi-Wei Ye, Lei Wen, Terance Gi-Wai Tsang, Jianli Cao, Jingjing Huang, Chris Chun-Yiu Chan, Kenn Ka-Heng Chik, Garnet Kwan-Yue Choi, Jian-Piao Cai, Feifei Yin, Hin Chu, Mifang Liang, Dong-Yan Jin, Kwok-Yung Yuen

**Affiliations:** 1 State Key Laboratory of Emerging Infectious Diseases, Li Ka Shing Faculty of Medicine, The University of Hong Kong, Pokfulam, Hong Kong, China; yuansf@hku.hk (S.Y.); hinchu@hku.hk (H.C.); 2 Department of Microbiology, Li Ka Shing Faculty of Medicine, The University of Hong Kong, Pokfulam, Hong Kong, China; zwye@hku.hk (Z.-W.Y.); wenlei07@hku.hk (L.W.); terancet@connect.hku.hk (T.G.-W.T.); caojl@hku.hk (J.C.); huangjj@hku.hk (J.H.); cyc415@hku.hk (C.C.-Y.C.); kchik929@connect.hku.hk (K.K.-H.C.); garnetchoi@yahoo.com (G.K.-Y.C.); caijuice@hku.hk (J.-P.C.); 3 Carol Yu Centre for Infection, Department of Microbiology, Li Ka Shing Faculty of Medicine, The University of Hong Kong, Pokfulam, Hong Kong, China; 4 Hainan Medical University-The University of Hong Kong Joint Laboratory of Tropical Infectious Diseases, Hainan Medical University, Haikou 571101, China, and The University of Hong Kong, Pokfulam, Hong Kong, China; yinfeifeiff@163.com; 5 Department of Pathogen Biology, Hainan Medical University, Haikou 571101, China; 6 Key Laboratory of Translational Tropical Medicine, Hainan Medical University, Haikou 571101, China; 7 Key Laboratory for Medical Virology and National Institute for Viral Disease Control and Prevention, Chinese Centre for Disease Control and Prevention, Beijing 102206, China; mifangl@vip.sina.com; 8 School of Biomedical Sciences, The University of Hong Kong, Pokfulam, Hong Kong, China; dyjin@hku.hk; 9 The Collaborative Innovation Center for Diagnosis and Treatment of Infectious Diseases, The University of Hong Kong, Pokfulam, Hong Kong, China

**Keywords:** antiviral, broxyquinoline, bunyavirales, eltrombopag, entry, hexachlorophene, Huaiyangshan banyangvirus, regorafenib, SFTSV, triclosan

## Abstract

Severe fever with thrombocytopenia syndrome virus (SFTSV) is an emerging tick-borne bunyavirus that causes severe disease in humans with case-fatality rates of up to 30%. There are currently very limited treatment options for SFTSV infection. We conducted a drug repurposing program by establishing a two-tier test system to rapidly screen a Food and Drug Administration- (FDA)-approved drug library for drug compounds with anti-SFTSV activity in vitro. We identified five drug compounds that inhibited SFTSV replication at low micromolar concentrations, including hexachlorophene, triclosan, regorafenib, eltrombopag, and broxyquinoline. Among them, hexachlorophene was the most potent with an IC_50_ of 1.3 ± 0.3 µM and a selectivity index of 18.7. Mechanistic studies suggested that hexachlorophene was a virus entry inhibitor, which impaired SFTSV entry into host cells by interfering with cell membrane fusion. Molecular docking analysis predicted that the binding of hexachlorophene with the hydrophobic pocket between domain I and domain III of the SFTSV Gc glycoprotein was highly stable. The novel antiviral activity and mechanism of hexachlorophene in this study would facilitate the use of hexachlorophene as a lead compound to develop more entry inhibitors with higher anti-SFTSV potency and lower toxicity.

## 1. Introduction

Severe fever with thrombocytopenia syndrome virus (SFTSV) is an emerging tick-borne virus in the genus *Banyangvirus*, family *Phenuiviridae*, order *Bunyavirales*. It was first identified in Huaiyangshan, a mountainous area in Huaiyang County in the Henan province of China [1]. Subsequently, the virus has also been isolated from infected humans, ticks, and mammals in Japan and South Korea. SFTSV is so named as it causes severe fever with thrombocytopenia syndrome (SFTS), an acute febrile illness characterized by high fever, thrombocytopenia, and hemorrhagic complications in infected humans. Patients with SFTS may also develop other clinical manifestations such as systemic upset, coma, slurred speech, gastrointestinal upset, hepatosplenomegaly, and lymphadenopathy, as well as abnormal laboratory findings including leukopenia, prolonged activated partial thromboplastin time, deranged liver function tests, proteinuria, hematuria, and elevated creatinine kinase and lactate dehydrogenate levels. The mortality rate of SFTS may be as high as 17% to 30% [1,2]. 

In addition to tick-borne transmission, non-vector-borne transmission of SFTSV has also been reported. A number of case clusters linked to nosocomial or intra-familial transmission have been described. While the majority of the secondary cases in these clusters were likely infected through unprotected direct contact with the index patients’ blood and/or bodily fluids with high viral loads, possible transmission of the virus through exposure to contaminated hospital environment surfaces and aerosols have also been reported recently [3,4,5,6].

Despite the clinical and public health importance of SFTSV, treatment options remain very limited. Ribavirin exhibits some inhibitory effects on SFTSV replication in vitro and in a type I interferon receptor-deficient mouse model, but it was not found to be effective in a retrospective cohort of patients [2,7]. Comparatively, favipiravir (T-705), an RNA-dependent RNA polymerase inhibitor with broad-spectrum antiviral activities, demonstrated higher in vitro and in vivo antiviral effects against SFTSV. However, favipiravir is not clinically approved or readily available in many countries affected by SFTSV, including China. To identify drug compounds that can be used to treat patients or reduce the transmission of SFTSV, especially in the hospital setting, we conducted a drug repurposing program by screening a Food and Drug Administration (FDA)-approved drug library consisting of 1528 drug compounds. We first established a robust two-tier (ELISA followed by viral load reduction assay) screening platform for SFTSV. Using this drug screening platform, we identified five drug compounds with anti-SFTSV activity in vitro, and characterized the anti-SFTSV mechanism of the most potent drug, hexachlorophene, as an entry inhibitor of SFTSV.

## 2. Materials and Methods

### 2.1. Virus, Cell Lines, and Drug Compounds

SFTSV HB29 strain (a gift from Dr. Benjamin Brennan, MRC-University of Glasgow Centre for Virus Research and Professor Mifang Liang, China CDC) was propagated in Vero cells and kept at −80°C in aliquots. Plaque forming unit (PFU) assay was performed to titrate the cultured virus. Vero (African green monkey kidney, ATCC, CCL-81) and Huh-7 (human hepatoma, JCRB, 0403) cells were maintained in Dulbecco’s modified eagle medium (DMEM, Gibco, CA, USA) culture medium supplemented with 10% heat-inactivated FBS (fetal bovine serum, Gibco), 50 U/mL penicillin, and 50 µg/mL streptomycin as previously described [8,9,10]. Upon virus infection, the cells were maintained in FBS-free medium with or without drug compounds. All experiments involving live SFTSV followed the approved standard operating procedures of the Biosafety Level 3 facility at the Department of Microbiology, The University of Hong Kong [11]. The FDA-approved drug library (Cat# HY-L022) and all the tested drug compounds were purchased from MedChem Express (Monmouth Junction, NJ, USA) unless otherwise specified. 

### 2.2. Cell Viability Assay and Cytopathic Effect (CPE) Inhibition Assay

The CellTiterGlo luminescent assay (Promega Corporation, Madison, WI, USA) was performed to detect the cytotoxicity of the selected drug compounds as previously described [12]. Briefly, Vero cells (4 × 10^4^ cells/well) were incubated with different concentrations of the individual compound for 72 h, followed by the addition of substrate and measurement of luminance 10 min later. The 50% cytotoxic concentrations (CC_50_) of the drug were calculated by Sigma plot (SPSS) in an Excel add-in ED50V10. To explore a suitable assay for FDA-approved drug library screening, the cytopathic effect (CPE) inhibition assay was also performed as previously described with slight modifications [13]. Briefly, Vero cells seeded in 96-well plates were infected with SFTSV for 1 h with different multiplicities of infection (MOI) of 1.00, 0.10, or 0.01, followed by phosphate buffered saline (PBS) wash and replacement of fresh DMEM medium containing 0.1% DMSO as the negative control or favipiravir (50 µg/mL) as the positive control. The cell viability of each well was determined on day 1, 3, and 5 post-infection (dpi) by the CellTiterGlo luminescent assay.

### 2.3. ELISA

ELISA was performed to determine the amount of viral nucleoprotein (NP) expression in the culture supernatant as previously described with modifications [14]. Briefly, 100 ng/well of rabbit-anti-SFTSV serum (Cat#PAB27171, Abnova, Taipei City, Taiwan) was coated in 96-well ELISA plates for overnight incubation at 4 °C, followed by blocking with 2.5% FBS plus 2.5% FBS in PBS with Tween 20 (Sigma-Aldrich, St. Louis, MO, USA) for 2 h at 37 °C. After washing, the culture supernatants collected at 1, 3, or 5 dpi were transferred to the ELISA plates accordingly (50 μL, incubated at room temperature for 2 h), followed by intensive wash and addition of 50 μL/well mouse-anti-SFTSV-NP serum (in-house preparation), the secondary goat-anti-mouse horseradish peroxidase (HRP) antibody (Invitrogen, Carlsbad, CA, USA), the 3,3′,5,5′-tetramethylbenzidine (TMB) solution (Invitrogen), and the stop solution (0.1M HCl). Subsequently, the optical density of each well was read at 450 nm (OD_450_) using VICTOR 3 multi-label plate reader (PerkinElmer, Inc., Waltham, MA, USA).

### 2.4. Viral Load Reduction Assay

Viral load reduction assay was performed as described previously with modifications [15,16]. Briefly, virus infection and drug treatment were first performed as described for the CPE inhibition assay. Then, the culture supernatants were collected at 1, 3, and 5 dpi, followed by total nucleic acid extraction. Quantitative reverse transcription-polymerase chain reaction (qRT-PCR) with previously established primers (forward 5′-GGGTCCCTGAAGGAGTTGTAAA-3′ and reverse 5′- TGCCTTCACCAAGACTATCAATGT -3′) and probe (FITC-TTCTGTCTTGCTGGCTCCGCGC-BHQ) targeting the S segment of the SFTSV genome was then performed using the Roche LightCycler Real-time PCR system [17].

### 2.5. Plaque Reduction Assay

Plaque reduction assay was performed as described previously with modifications [18]. Briefly, Vero cells were seeded at 2 × 10^5^ cells/well in 24-well tissue culture plates on the day before the assay was performed. After 24 h of incubation, 60–80 PFUs of SFTSV were added to the cell monolayer with or without the addition of selected drug compounds, and the plates were further incubated for 1 h at 37 °C in 5% CO_2_ before removal of unbound viral particles by aspiration of the media and washing once with DMEM. The cell monolayers were then overlaid with media containing 1% low melting agarose (Cambrex Corporation, East Rutherford, NJ, USA) in 2% FBS–DMEM and appropriate concentrations of drug compounds, inverted, and incubated as stated above for another 8 days. Next, the wells were fixed with 10% formaldehyde (BDH, Merck, Darmstadt, Germany) overnight. After removal of the agarose plugs, the cell monolayers were stained with 0.7% crystal violet (BDH, Merck), and the number of plaques was counted. The percentage of plaque inhibition relative to the control (i.e., without the addition of a drug compound) wells were determined for each drug concentration. The half maximal inhibitory concentration (IC_50_) was calculated using Sigma plot (SPSS) in an Excel add-in ED50V10.

### 2.6. Virus Fusion Assay 

To determine if hexachlorophene inhibits the cell membrane fusion of SFTSV, syncytium formation of virus-infected cells was determined under low-pH exposure as previously described [19]. Briefly, Vero cells were infected with SFTSV at 0.01 MOI. At 24 hpi, the cells were rinsed once with PBS and then incubated with citrate-phosphate buffer (0.1 M citric acid and 0.2 M sodium dihydrogen orthophosphate, Sigma-Aldrich, St. Louis, MO, USA) adjusted to pH 5.0 for 2 min. The citrate-phosphate buffer was then replaced with 10% FBS–DMEM with or without hexachlorophene treatment. After 5 h, cell fusion within monolayers was observed under a phase-contrast microscope.

### 2.7. Molecular Docking Analysis

The 3D structure of hexachlorophene (ID code: DB00756) was downloaded from the Drugbank database (https://www.drugbank.ca/). The crystal structure of SFTSV Gc protein (PDB code: 5G47) was used to build up the docking receptor [20]. Bond orders were assigned, hydrogens were added, and cap termini were included with the Protein Preparation Wizard module as implemented in Maestro [21]. Protonation states of side chains were predicted by using PROPKA3.1 server [22]. Partial charges over all atoms were finally assigned within the AMBER99 force field scheme as implemented in AmberTools. Ligand binding sites were predicted with Metapocket2.0 server [23]. Ligand-receptor docking was performed with LeadFinder version 1804 [24]. The top-ranked pose was visualized with Pymol. Intermolecular interactions were visualized with Ligplot+ [25].

## 3. Results

### 3.1. Establishment of a Robust Antiviral Screening Platform for SFTSV

**Figure 1:**
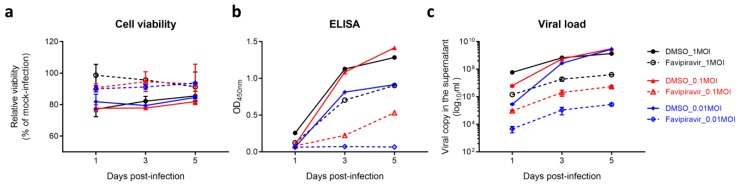
Optimization and comparison of biochemical assays for Food and Drug Administration- (FDA) approved drug compound library screening. Vero cells seeded in 96-well plate were infected with severe fever with thrombocytopenia syndrome virus (SFTSV) for 1 h with the multiplicities of infection (MOIs) indicated (1, 0.1 and 0.01), followed by phosphate buffered saline (PBS) wash and replacement of fresh Dulbecco’s modified eagle medium (DMEM) containing 0.1% DMSO (negative control) or favipiravir (50 µg/mL, positive control). (**a**) Cell viability of each well was determined on day 1, 3, and 5 post-infection, which was normalized by that of the mock-infected cells. (**b**) Cell culture supernatant was collected at the indicated time points and applied for ELISA to measure the SFTSV-nucleoprotein (NP) protein intensity. (**c**) Alternatively, viral copy in the supernatant was determined by quantitative RT-PCR (qRT-PCR). The experiments were carried out in triplicate. The results are shown as mean ± standard deviation.

To establish a sensitive assay for anti-SFTSV drug screening, we compared the signal dynamic range and screening window of three assays (i.e., CPE inhibition assay, ELISA, and viral load reduction assay). The cell viability, SFTSV-NP protein expression, and the S-segment viral genome copy number in the culture supernatant were monitored at 1, 3, and 5 dpi, respectively. Favipiravir was used as a positive control in all three assays [7]. As shown in Figure 1a, differences of cell viability between the favipiravir-treated and the DMSO-treated groups were less than 20%, indicating that the CPE inhibition assay was not a sensitive assay for drug screening when favipiravir was used as a positive control. Next, the culture supernatants were subjected to ELISA and qRT-PCR analyses. As shown in Figure 1b, >10-fold and >15-fold read-outs between the favipiravir-treated and the DMSO-treated groups were detected with 0.01 MOI at 3 dpi and 5 dpi, respectively, by ELISA. In the viral load reduction assay, there was approximately 4-log_10_ difference in SFTSV RNA load between the two groups at the 3 dpi and 5 dpi (Figure 1c). These data suggested that both the ELISA and the viral load reduction assay were sensitive drug screening assays for SFTSV. Considering the cost-effectiveness of the two assays and the need to balance between maximum drug efficacy and drug half-life (t_1/2_), drug compound library screening was then performed using ELISA with 0.01 MOI of SFTSV as virus inoculum and the culture supernatant being collected at 3 dpi.

### 3.2. Identification of Anti-SFTSV Drug Compounds

**Figure 2:**
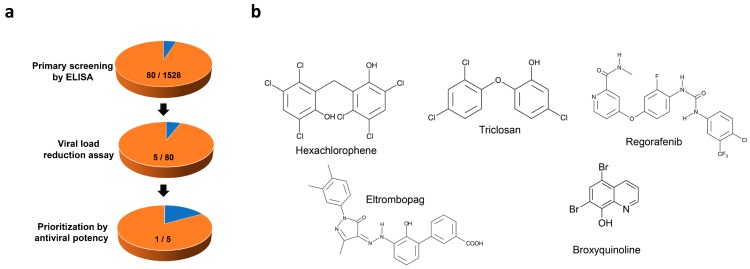
Identification of anti-SFTSV drug compounds. (**a**) Screening pipeline and attrition rates of compounds from primary screening by ELISA, secondary screening by viral load reduction assay, followed by prioritization by IC_99_. (**b**) Shown are chemical structures of five selected drugs that show dose-dependent inhibition of SFTSV replication.

An FDA-approved drug library with 1528 drug compounds was screened using the optimized conditions with the concentration of each drug set at 10 µM. ELISA was utilized for primary screening, followed by viral reduction assay as a secondary validation assay. Among the 1528 drug compounds, the top 80 primary hits with >50% reduction of absorbance signal in ELISA were subjected to viral load reduction assay (fixed drug compound concentration of 10µM) to further select compounds that can inhibit SFTSV replication in a dose-dependent manner. Drug compounds with obvious cytotoxicity at ≤10 µM were excluded. Five drug compounds, namely hexachlorophene, triclosan, regorafenib, eltrombopag, and broxyquinoline, were identified as anti-SFTSV drug compounds after this two-step screening (Figure 2b). These five drug compounds were then prioritized by their antiviral potency (i.e., IC_50_ and IC_99_) (Table 1). Hexachlorophene was selected for further characterization, as it had the lowest IC_50_ (1.3 ± 0.3 µM) and the highest selectivity index (CC_50_/IC_50_, 18.7) among the five drug compounds in the viral load reduction assay.

### 3.3. Ant-SFTSV Activity of Hexachlorophene In Vitro 

**Figure 3:**
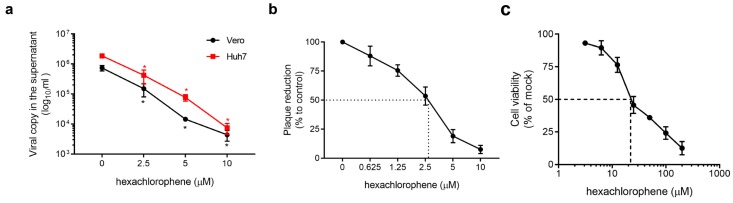
Evaluation of the in vitro anti-SFTSV activity of hexachlorophene. (**a**) SFTSV viral load reduction quantified by qRT-PCR in Vero and Huh 7 cells at 72 hpi (0.01 MOI) with hexachlorophene. (**b**) Half maximal inhibitory concentration (IC_50_) of hexachlorophene is around 2.6 ± 0.14 µM as determined by plaque reduction assay in Vero cells. (**c**) Cell cytotoxicity assay of hexachlorophene in Vero cells as determined at 72 hpi. All experiments were performed in triplicates. * denotes *p* < 0.05 (compared to the DMSO control group by one-way ANOVA). Data are presented as mean values ± standard deviation (error bars).

The anti-SFTSV activity of hexachlorophene was further validated by viral load reduction assay in different cell lines and plaque reduction assay. Dose-dependent reduction in the viral RNA load was observed in the culture supernatant of hexachlorophene-treated Vero and Huh-7 cells with an IC_50_ of 1.3 ± 0.3 µM (Figure 3a). At 10 µM, hexachlorophene reduced the viral RNA load by ≥2-log_10_ at 72 hpi. In the plaque reduction assay, hexachlorophene achieved 100% plaque reduction at ~10 µM, with an IC_50_ of 2.6 ± 0.14 µM (Figure 3b). In the absence of SFTSV infection, hexachlorophene exhibited a CC_50_ of 24.3 ± 3.2 µM at 72 hpi in Vero cells (Figure 3c).

### 3.4. Hexachlorophene Interferes with SFTSV Entry into Cells

**Figure 4:**
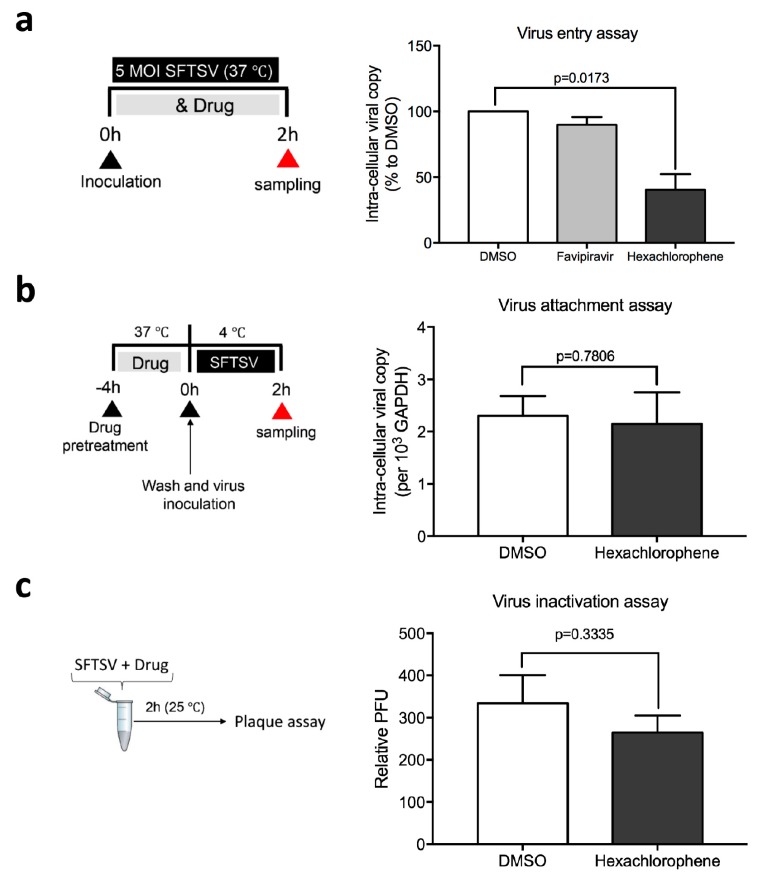
Hexachlorophene interferes with SFTSV entry without inhibiting viral attachment to host cells or inactivating the virions. (**a**) SFTSV entry assay. Vero cells were infected with the mixture of SFTSV (MOI = 5.0) and indicated drug for 2 h, followed by intensive wash and detection of intracellular SFTSV viral RNA load by qRT-PCR assays. Favipiravir (T-705), a known virus polymerase inhibitor, was used as the negative control. (**b**) SFTSV attachment assay. Vero cells were pre-treated by hexachlorophene for 4 h, followed by intensive wash and shift to 4 °C incubate with SFTSV (MOI = 5.0). After 2 h, the infectious inoculum was removed, cells were washed, and the intra-cellular viral RNA load was determined by qRT-PCR. (**c**) SFTSV inactivation assay. SFTSV was incubated with 10 µM hexachlorophene for 2 h, followed by standard plaque assay from diluting the mixture for 1000 fold (i.e., the remaining concentration of hexachlorophene was below its IC_50_). All experiments were performed in triplicates. Data are presented as mean values ± standard deviations. P value was calculated by Student’s *t*-test (compared with the DMSO group).

To differentiate whether the entry or the post-entry phases of the SFTSV replication cycle were interrupted by hexachlorophene, we performed a virus entry assay by exposing SFTSV-infected cells to hexachlorophene during the virus entry step, followed by quantification of the intracellular SFTSV viral RNA load at 2 hpi. As shown in Figure 4a, hexachlorophene or DMSO was co-mixed with SFTSV (5 MOI) and incubated with Vero cells for 2 h. Significantly lower viral RNA load was detected in the cells co-mixed with hexachlorophene (*p* = 0.0173) than those co-mixed with DMSO. Expectedly, there was no statistically significant difference between the DMSO and favipiravir groups, as the latter is a viral polymerase inhibitor (Figure 4a). The result indicated that hexachlorophene treatment interfered with SFTSV entry. To further dissect the anti-SFTSV mechanism of hexachlorophene, we performed virus attachment and inactivation assays to investigate whether the drug inhibited virus attachment to the host cell surface or directly inactivated the viral particles by binding to the viral envelope. As shown in Figure 4b, no significant (*p* = 0.7806) inhibitory activity was observed when hexachlorophene was added to Vero cells pretreated with the drug compound (−4 to 0 hpi), which suggested that virus attachment to the host cell surface was not affected. Virus infectivity was also not significantly (*p* = 0.3335) impaired when SFTSV was pre-incubated with hexachlorophene for 2 h, followed by the detection of plaque formation when hexachlorophene concentrations were <0.1 µM (Figure 4c).

### 3.5. Hexachlorophene Inhibits Membrane Fusion of SFTSV

**Figure 5:**
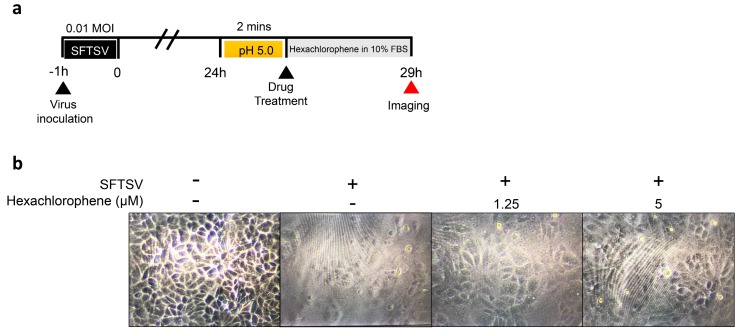
Hexachlorophene inhibits membrane fusion of SFTSV. (**a**) Schematic representation of the experimental procedures. Vero cells were infected with SFTSV (MOI = 0.01) for 1 h. At 24 hpi, the cells were treated with citrate-phosphate buffer adjusted to the pH 5.0 for 2 min, washed, and then replaced with DMEM containing 10% fetal bovine serum (FBS) and different concentrations of hexachlorophene. Syncytium formations were determined by microscopic examination at 5 h after drug addtion. (**b**) Syncytium formation of SFTSV-infected Vero cells with or without hexachlorophene treatment. Mock-infected Vero cells (left) were included as the negative control (magnification 80×).

The fusion of a virus-infected cell with neighboring cells leads to the formation of multi-nucleate enlarged cells and syncytia [19,26]. This event is induced by surface expression of viral fusion proteins that are fusogenic directly at the host cell membrane. Only viruses that are able to directly fuse at the cellular surface without the need of endocytosis induce syncytium formation. Syncytium formation of SFTSV-infected cells can be triggered by low pH buffer [19]. Since hexachlorophene interfered with SFTSV entry without inhibiting viral attachment to host cells or inactivating the virions (Figure 4), we therefore asked if hexachlorophene inhibited SFTSV membrane fusion. To this end, we first treated SFTSV-infected cells in citrate-phosphate buffer adjusted to pH 5.0. Then, the cells were treated with hexachlorophene (5.0 µM, 2.5 µM, or 0 µM) (Figure 5a). As shown in Figure 5b, syncytium formation in SFTSV-infected cells was dramatically inhibited by hexachlorophene in a dose-dependent manner, indicating that SFTSV-induced cell fusion was impaired. 

### 3.6. Hexachlorophene is Predicted to Occupy the SFTSV Gc Hydrophobic Pocket

**Figure 6:**
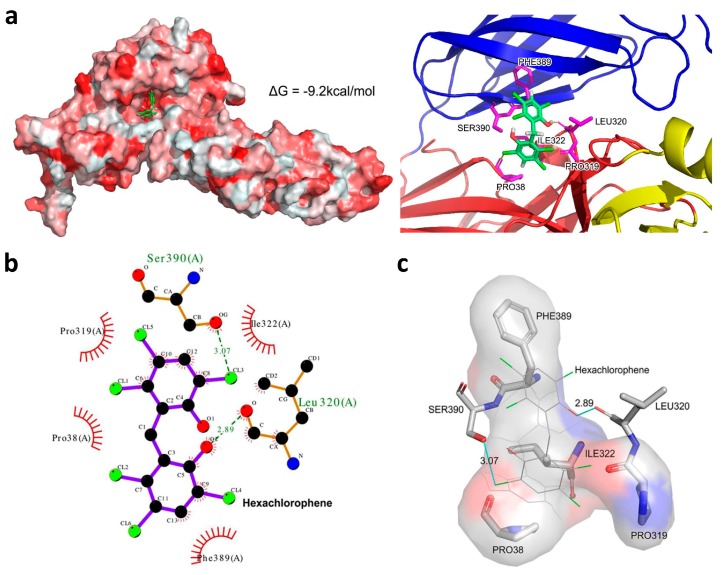
Docking model between hexachlorophene and the SFTSV Gc glycoprotein. (**a**) Left: top-ranked docking pose predicting the binding between hexachlorophene and the deep hydrophobic pocket of the SFTSV Gc glycoprotein. Hydrophobicity is highlighted in red. Right: the SFTSV Gc glycoprotein domains I, II, and III are highlighted in red, yellow, and blue, respectively. (**b**) 2D and (**c**) 3D intermolecular interaction showing hydrogen bonding, halogen bonding, and hydrophobic interactions between hexachlorophene and Gc protein. Hydrogen and halogen bonds are indicated in blue lines, and the distances are also labeled.

Viral envelope proteins are known to fuse with plasma or endosomal membranes to allow the release of the viral genome. The SFTSV M segment encodes the two viral envelope glycoproteins (GPs), Gn and Gc. The N-terminal, Gn, is responsible for receptor binding to the cell surface while the C-terminal, Gc, is a fusion protein that facilitates virus entry [27]. To characterize the relative binding affinity and intermolecular interactions between hexachlorophene and the SFTSV Gc glycoprotein, molecular docking was performed to predict the binding pose of the drug. As shown in Figure 6a, hexachlorophene was predicted to bind with the deep hydrophobic pocket between domain I and domain III of the SFTSV Gc glycoprotein with a relative binding free energy ΔG output of −9.2 kcal/mol [20]. Broad areas of hydrophobic interactions between the ligand and the protein were predicted to occur, and the hydrogen and halogen bonds were predicted to stabilize the binding pose (Figure 6b,c). Overall, these findings suggested that the binding of hexachlorophene to the hydrophobic pocket at the SFTSV Gc glycoprotein was highly stable.

## 4. Discussion

As exemplified by the recent epidemics caused by severe acute respiratory syndrome and Middle East respiratory syndrome coronaviruses, Ebola virus, and Zika virus, the de novo development of new antiviral treatments invariably lags behind the rapid progression of emerging viral outbreaks [28,29,30]. Thus, drug repurposing programs have been increasingly conducted to find clinically approved drugs with in vitro and/or in vivo activity against emerging viruses [31]. The candidate drug compounds identified in these drug repurposing programs have the advantages of being clinically available with known pharmacokinetics, pharmacodynamics, and side effect profiles. This information would facilitate more rapid development of the repurposed drug compounds into clinical use. SFTSV is an emerging tick-borne bunyavirus that causes severe disease in infected humans with very limited antiviral treatment options. In this study, we established a robust two-tier drug screening platform and then used it to screen a drug library consisting 1528 FDA-approved drug compounds to successfully identify five drugs that were able to inhibit SFTSV replication at low micromolar levels. These included antibacterial and antifungal disinfectants (hexachlorophene and triclosan), multi-kinase inhibitor for the treatment of advanced solid organ tumors (regorafenib), small molecule agonist of the c-mpl receptor for the treatment of immune thrombocytopenic purpura and aplastic anemia (eltrombopag), and antiprotozoal agent (broxyquinoline).

Among these five drugs, hexachlorophene demonstrated the highest antiviral potency in vitro. Hexachlorophene is an organochlorine compound that was widely used as a topical antibacterial and antifungal disinfectant and surgical scrub, as well as in agriculture as a bactericide, fungicide, and pesticide [32]. Moreover, hexachlorophene also exhibited antiviral activity against various RNA and DNA viruses. Disinfectants containing hexachlorophene exhibited antiviral activity against rhinovirus (in combination with ethyl alcohol) and rotavirus (0.75% hexachlorophene or 0.1% hexachlorophene with 70% isopropylalcohol) [33,34]. Hexachlorophene inhibited the 3C-like protease activity of severe acute respiratory syndrome coronavirus and the in vitro replication of other coronaviruses, including murine hepatitis virus, bovine coronavirus, and human enteric coronavirus [35,36]. Hexachlorophene also exhibited antiviral activity against polyomaviruses (BK virus and Simian Virus 40) by inhibition of the large tumor antigen’s ATPase activity of these viruses [37].

In the present study, we further expanded the spectrum of antiviral activity and described a novel antiviral mechanism of hexachlorophene. We showed that hexachlorophene potently inhibited SFTSV replication, as evidenced by significant (>2-log_10_) reduction of viral RNA load and 100% plaque reduction at a drug concentration of 10 µM, which was below its CC_50_. The IC_50_ of hexachlorophene against SFTSV (1.3–2.6 µM) was lower than that the other anti-SFTSV drug compounds reported thus far (caffeic acid, 180 µM; ribavirin, 40.1 µM; favipiravir, 25.0 µM; amodiaquine, 19.1 µM; and 2′-fluoro-2′-deoxycytidine, 3.7 µM) [38,39,40]. Using a combination of virus entry, attachment, inactivation, and membrane fusion assays, we showed that hexachlorophene interfered with virus entry and virus-induced cell fusion without affecting virus attachment to host cells or inactivating the virions. Molecular docking predicted that hexachlorophene bound stably with the deep hydrophobic pocket between domain I and domain III of the SFTSV Gc glycoprotein, providing insights into the structural interactions between hexachlorophene and SFTSV that explain the drug’s in vitro antiviral activity.

The novel findings in this study have a number of important implications. First, we showed that our two-tier drug screening platform is a robust system that can be utilized to identify anti-SFTSV drug compounds rapidly. This system could be used to screen larger chemical libraries containing more drug compounds to find additional treatment options for SFTSV. Second, the novel antiviral activity and mechanism of hexachlorophene in this study would facilitate the use of hexachlorophene as a lead compound to developing more entry inhibitors with higher anti-SFTSV potency and lower toxicity. The antiviral activity and mechanism of the other identified hit compounds by our two-tier screening system should be further characterized in future studies. Finally, the use of hexachlorophene as a disinfectant should be considered to reduce the risk of nosocomial outbreaks of SFTSV associated with contaminated environmental surfaces.

## Figures and Tables

**Table 1:** Anti-SFTSV activity and cytotoxicity of five selected drug compounds.

Compound	IC_99_ (µM)	IC_50_ (µM)	CC_50_ (µM)	Selectivity Index
Hexachlorophene	7.5 ± 1.2	1.3 ± 0.3	24.3 ± 3.2	18.7
Triclosan	8.5 ± 2.1	3.2 ± 0.4	17.7 ± 2.9	5.5
Regorafenib	11.3 ± 0.5	4.5 ± 0.5	31.3 ± 0.5	7.0
Eltrombopag	10.3 ± 3.4	4.1 ± 0.2	18.4 ± 0.2	4.5
Broxyquinoline	16.3 ± 4.3	5.8 ± 1.3	36.4 ± 5.5	6.3
